# Identifying adoption and usability factors of locator devices for persons living with dementia

**DOI:** 10.1177/14713012211065381

**Published:** 2021-12-29

**Authors:** Noelannah Neubauer, Christa Spenrath, Serrina Philip, Christine Daum, Lili Liu, Antonio Miguel-Cruz

**Affiliations:** 153482 University of Waterloo, Canada; Department of Occupational Therapy, 70412 University of Alberta, Canada; Department of Occupational Therapy, 70412 University of Alberta, Canada; 153482 University of Waterloo, Canada; 153482 University of Waterloo, Canada; Department of Occupational Therapy, 70412 University of Alberta, Canada; 153482 University of Waterloo, Canada; 153482 University of Waterloo, Canada; Department of Occupational Therapy, 70412 University of Alberta, Canada; Glenrose Rehabilitation Research, Innovation & Technology (GRRIT) Hub, 60351 Glenrose Rehabilitation Hospital, Canada

**Keywords:** dementia, locator technologies, usability, acceptability, lost persons

## Abstract

A growing number of Canadians live with dementia. Strategies to reduce the risks of getting lost include physical barriers, restraints and medications. However, these strategies can restrict one’s participation in meaningful activities and reduce quality of life. Locator devices can be used to manage safety risks while also supporting engagement and independence among persons living with dementia. As more locator devices become available on the market, adoption rates would be affected by certain factors. There is no clear, standardized approach to identify the factors that have an influence on the acceptance and usability of locator devices for persons with dementia and their care partners. This project aimed to identify factors related to acceptance and usability of locator devices that are important to individuals with dementia, their care partners, service providers and technology developers. Qualitative description and conventional content analysis guided our approach. We conducted 5 focus groups with 21 participants. Trustworthiness strategies included multiple data sources, data verification for accuracy and peer debrief. Five overarching factors emerged as critical aspects in the acceptance and usability of locator devices. These factors were inclusivity, simplicity, features, physical properties and ethics. Participants thought that locator devices do not adequately consider privacy and stigma. Therefore, the acceptance and usability of locator devices could be enhanced if privacy and stigma are addressed. The factors identified will inform the creation of an acceptance and usability scale for locator devices used by persons living with dementia, their care partners and service providers.

## Introduction

Dementia continues to prevail as a significant public health concern with approximately 50 million cases worldwide (World Health Organization, 2020). One of the behaviours associated with dementia that presents a major concern for service providers and care partners are lost incidents (Eichler et al., 2016; Neubauer, Lapierre, et al., 2018). Lost incidents occur when a person living with dementia becomes unable to recognize places and has difficulty in wayfinding (Rowe & Bennett, 2003). Approximately 60% of persons living with dementia will become lost at least once during the progression of their disease (Hadwen et al., 2017; Mangini & Wick, 2017; Rowe & Bennett, 2003; Tilly, 2015). Adverse outcomes associated with getting lost include injury, death (Niemeijer, 2015; Rowe & Bennett, 2003), and for some, placement in a care facility (Furumiya & Hashimoto, 2014; White & Montgomery, 2014). Lost incidents can also lead to increased levels of stress among individuals with dementia and care partners (Neubauer, Hillier, et al., 2018; Shalev Greene et al., 2019).

Traditionally, physical barriers, restraints and medications have been used to manage the risks of getting lost (Hermans et al., 2007; Price et al., 2001). These approaches limit the potential therapeutic benefits of walking as a means expressing unmet needs (Moser, 2019) or engaging in one’s community (Brittain et al., 2017). As a result, alternative strategies to mitigate the risks associated with getting lost would benefit individuals at risk. The rapid growth of contemporary technologies, such as information and communication technologies, has created new avenues for promoting health interventions (Rosenberg et al., 2012). Locator devices, for example, may promote safe walking while simultaneously enhancing a balance between an individual’s autonomy and safety (Moyle, 2019; Wherton et al., 2019).

Although technological interventions, such a locator devices, can play a role in enhancing the quality of life for persons with dementia, the adoption rate of these devices are relatively low (Demers et al., 2016; Miguel Cruz et al., 2020; O’Sullivan et al., 2017). Like assistive technologies, the abandonment of these devices is a concern; where privacy, cost and device complexity can pose barriers to their use among older adults (Demers et al., 2016; Peek et al., 2016). The legal repercussions of technologies that collect, store and upload data have not been considered or addressed (Moyle, 2019). Locator devices, for example, place users at risk of their information being publicised (Moyle, 2019), which in turn can put vulnerable users, such as persons with cognitive decline, at greater risk for elder abuse (Jotterand et al., 2019).

While technology acceptance has become a growing field in health research (Miguel Cruz et al., 2020), no standardized scale exists for assessing the technology acceptance and usability of locator devices for persons who have dementia and their care partners. Such a scale is needed as more locator devices become available on the market. In addition, there are conflicting perspectives regarding the balance between safety and autonomy among persons with dementia who are at risk or have already gone missing, and those that are responsible for their care (Cooper et al., 2019).

The identification of factors used in the development of an acceptance and usability scale for locator devices requires involvement of individuals living dementia, and stakeholders involved in their care such as service providers, care partners and members of industry. This scale would have the potential to improve the number of locator devices that are accepted and used. It could do this by helping persons with dementia and care partners find the best device that suit their needs, thereby enhancing acceptance and adoption. Such a scale would also inform developers and designers to create user friendly products. The purpose of this study was to identify factors related to acceptance and usability of locator devices that are important to persons living with dementia, their care partners, service providers and technology developers.

## Method

### Design

We used qualitative description (Sandelowski, 2000) to understand the factors related to the acceptance and usability of locator devices. Qualitative description is appropriate when seeking to provide a descriptive summary of the experiences and opinions of a group of people in relation to a phenomenon (Kim et al., 2017; Neergaard et al., 2009).

### Participants, sample size and recruitment

To enhance the richness and quality of the data collected, a snowball sampling method (Patton, 2002) was used to identify key informants. Participants with professional or lived experience of locator technologies were recruited through our professional networks as they could provide insight into the factors that have an influence on the acceptance and usability of locator devices. To ensure we appropriately reflected the diversity of experiences and involvement in this research area, we intentionally selected the following four stakeholder groups: service providers (e.g. occupational therapists, dementia educators, and social workers), technology developers, care partners of persons living with dementia, and persons living with dementia.

All participants were required to speak English and be over the age of 18 years. A total of five focus groups were conducted, that is, one focus group was service providers, one comprised of technology developers, two focus groups involved only care partners, and one was for persons living with dementia. Each stakeholder group also had specific inclusion criteria. Persons living with dementia were included in the study if they had a mild cognitive impairment or mild dementia to ensure that they could respond to the questions during the focus group. The degree of cognitive impairment was determined using a teach-back method (Holtz & Byrdsong, 2020) during the consent process. Teach-back uses open-ended questions and asks respondents to answer in their own words. Questions included: Can you tell me about the procedures you would be asked to complete if you participate in this study? What are some of the risks or problems you may experience from your participation in this study and how do you think they will affect you? What would you do if you wish to withdraw from the study? Care partners were required to have previous or current experience in caring for a person with dementia. Service providers were required to have at least 2 years of experience providing services for persons living with dementia. Lastly, developers were required to have experience developing locator devices for dementia populations. Exclusion criteria included: people unfamiliar with locator devices; people with severe physical, visual or hearing limitations that could not be corrected with the use of an assistive device; people with mental or cognitive impairments who were unable to provide informed consent. Ethics approval was obtained from the University of Alberta Research Ethics Board and in accordance with the Declaration of Helsinki. Written informed consent was obtained from participants prior to participation in this study.

### Data collection and preparation

We used focus groups to ensure the collection of a broad range of perspectives (Sandelowski, 2000) regarding the necessary features of devices. Focus groups encourage participants to expand and respond to the ideas of others (Powell & Single, 1996). Focus groups took place using Zoom videoconference (Zoom Video Communications, Inc., headquartered in San Jose, California) to facilitate recruitment in Canada and in the United Kingdom, and to enable data collection despite lockdown restrictions due to the SARS-CoV-2 (or COVID-19) pandemic.

Five focus groups took place over the course of 5 months. All participants and facilitators had their camera and microphones turned on for the duration of the focus groups. Each focus group began with the researchers presenting the study background, purpose and findings of literature review conducted by the team (Miguel Cruz et al., 2020). A general summary of findings from the previous focus groups were shared at the beginning of each subsequent focus group. Participants were told that focus groups with other stakeholders were convened and that facilitators would seek participants’ perspectives based on their experience. Participants were also told that facilitators would ask questions and seek clarification when differences emerged between stakeholder groups.

Guiding questions for all focus groups included: What are your experiences working with locator devices? From your experience, what worked and did not work for yourself/your clients/the person you care for as it relates to locator devices? From the factors discussed so far, what are we missing? What other perspectives do we need to consider when looking at the factors that influence the acceptance and usability of locator devices? Participants who had dementia were asked an additional guiding question at the beginning of their group regarding any instances they had gotten lost and what they did to find their way back.

One facilitator led the discussion and a second and third facilitator created a record of factors generated by participants as well as notable observations (technical challenges, interruptions, interactions between participants, body language). These were always the same facilitators to ensure consistency. To verify the preliminary results with participants (Guba, 1981), factors generated by the group were screen shared with participants at the end of each focus group. This was done to allow participants to correct any misunderstandings, further expand on ideas and add factors if any were missed.

### Data analysis

Focus groups were audio recorded and transcribed verbatim by a professional transcription service. Transcripts and field notes were read and reviewed multiple times to ensure accuracy (Poland, 1995). Conventional content analysis (Hsieh & Shannon, 2005) guided our data analysis. Transcripts were annotated and coded based on their content. Codes were then organized into categories. After analysing each focus group, we compared the findings across the different stakeholder groups. A role-ordered matrix was used to compare and contrast responses. Data regarding each focus group was summarized in a table and cross referenced.

We adopted verification strategies proposed by Morse et al. (2002) to enhance rigour during data collection and analysis. Methodological coherence, sampling adequacy, concurrent data collection and analysis, and thinking theoretically were adopted. These were supplemented with aspects of trustworthiness strategies described by Lincoln et al., (1985), specifically verification for data accuracy, peer debrief and keeping an audit trail.

## Findings

### Participants

The sample comprised of service providers (*n* = 6), persons living with dementia (*n* = 5), care partners (*n* = 2, *n* = 3) and technology developers (*n* = 5). Focus groups ranged in length from 79 min to 110 min with an average duration of 92 min. Participants were from three countries (Canada, England, Ireland) and four Canadian provinces (British Columbia, Alberta, Ontario, Nova Scotia). See Table 1 for participant demographics.

**Table 1.** Participant characteristics.

Stakeholder	Previous/current occupation, role in occupation setting	Type of dementia	Other relevant	Location	Experience with GPS devices/Getting lost
Service provider	Public health (1); Occupational therapist (1); Adult and continuing education (1); Social worker (2); Gerontologist	N/A	N/A (4), care partner for parents (1), care partner for stepfather (1)	British Columbia (1); Alberta (2); Ontario (2); Ireland (1)	N/A
Developer	Industry leader (1), engineer (2), Computing scientist (1), technology Service manager (1)	N/A	Focus on non-intrusive monitoring for seniors (5), designs remote caregiving solutions (4), develops intelligent navigation systems (1), builds assistive technologies (4)	England (1); Nova Scotia (1); Ontario (3)	N/A
Care partners	N/A (2), engineer (1), Nurse (1)	Unspecified (3), Alzheimer’s (1) mixed and Alzheimer’s (1)	Care partner for husband (3), care partner for wife (1), care partner for sister and brother-in-law (1)	Ontario (5)	Unknown (1), uses phone GPS (2), Caretrak (1)
People living with Dementia	Dementia advocacy groups (5), Assistant executive housekeeper (1)	Frontal temporal (1), Alzheimer’s with vascular components (1), vascular dementia (2), Mild cognitive impairment (1)	Lives with husband (1); Lives alone (4)	British Columbia (2), Alberta (1), Ontario (2)	Has gotten lost (5)

Numbers in brackets represent the number of participants in each focus group.

### Factors in choosing a device

**Figure 1.**
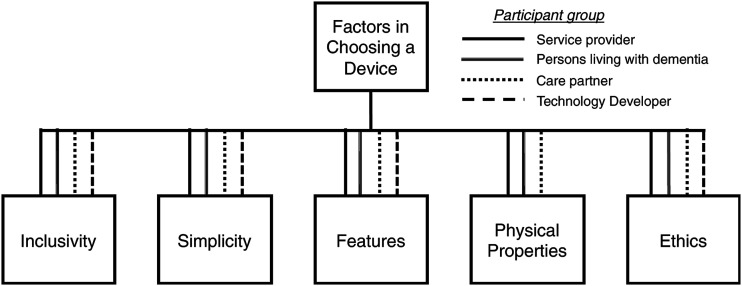
Overall factors that have an influence on the usability of locator devices for people with dementia, by participant group.

The data generated five main factors including: inclusivity, simplicity, features, aesthetic appeal and ethics (Figure 1). All five factors were discussed by all stakeholder groups with the exception of aesthetic appeal, which was not identified by the technology developers. These five factors were all broken down further into subcategories as described below.

#### Inclusivity

**Figure 2.**
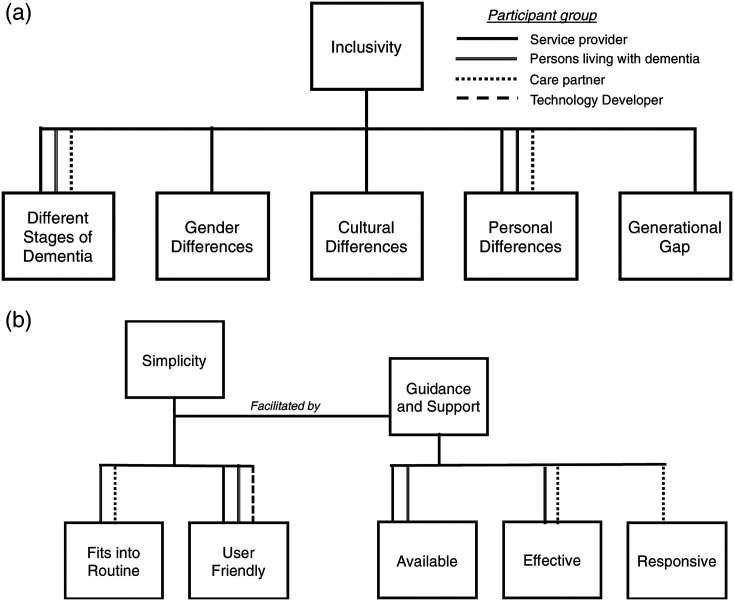
Factors that encompass: (a) inclusivity, and (b) simplicity of locator devices for people with dementia, by participant group.

Inclusivity, within the context of this study, is defined as aiming to provide access to locator device resources for persons living with dementia. An inclusive device accounts for a variety of differences between individuals and allows them to all utilize the device equally to assist in mitigating the risks associated with getting lost. To support the inclusivity of these devices, participants agreed that the voices and differing needs of persons living with dementia should be considered during technology development and evaluation of locator devices. Subfactors of inclusivity identified by participants included the device accounting for the different stages of dementia, multiple languages, gender (i.e. expectations on the type of wearable worn such as jewellery) cultural and personal differences and generation gaps (Figure 2(a)). Gender differences as described by one of the service providers included the following:

“Gender can be a piece because our outdoor spaces, and our social networks are different. I remember one carer talking about how she worried it [the GPS device] could be emasculating for her to wear one of these devices. So, it’s just interesting, those cultural pieces are quite nuanced, and always gendered”. - Service Provider #4

##### Stages of Dementia

Service providers, care partners and persons with dementia indicated that it is important for a device to account for the stages of dementia, as each stage presents differently and creates different needs. For example, in the early stages of dementia, a person living with dementia may want to have a device that can provide directions to their destination. In the later stages of dementia, however, when they are less engaged in their community, the sole focus of the device may be more focused on the ability for the care partner to track the individual with dementia. A service provider also suggested that it would be helpful for a device to be integrated in the early stages of a person’s diagnosis of dementia to assist in the adoption of the device during the later stages:

“I’ve met people that are wanting to start using [a locator device] now because they’re in early stage and they want to get used to something so that down the line, they’re ready.” – Service provider #3

##### Gender, culture and personal differences

Service providers also suggested that locator devices should account for gender differences because this influences interaction with space and social networks which may, in turn, influence interaction with locator devices. Culture may also influence preferences, habits, roles and values and this may affect a person’s use of a locator device. For example, certain cultures may be more or less accepting to the level of privacy between the person with dementia and care partner while the person with dementia is wearing a locator device. For the device to be usable by all individuals, it should account for how gender and culture may interact with the adoption of locator devices. Personal differences, such as medical conditions (e.g. allergies, cardiac challenges resulting in a pacemaker) may also have an influence on the adoption of locator devices as they may be contraindicated or cause difficulties for the user.

“I can’t even wear a watch. If I pick up something plastic, I get a red mark and it swells so I’m really caught between a rock and a hard place, and I’ve tried many of these things but haven’t been able to wear them.” – Person with dementia #1

##### Generation gap

A difference currently exists between the attitudes and technology usage of different age groups, which, within the context of this study, can be identified as a generation gap. With this difference in attitudes comes a difference in acceptability and usage of including locator devices. As noted by service providers, some older adults may have limited experience using technologies, such as a smartphone or computer. Therefore, devices that require the use of these platforms, may be challenging.

#### Simplicity

Simplicity was identified by all stakeholder groups and was described as ensuring that a device fits into an individual’s routine, is user friendly (Figure 2(b)).

##### Fits into routine

For a device to be simple, care partners and person with dementia indicated that it should fit seamlessly into wearers’ established routines. The device could be embedded in an item that the person with dementia already uses, such as a watch. If a device is incorporated into a wearer’s routine, such as a GPS insole in one of many pairs of shoes, this can decrease the chance that user forgets to wear it or carry it with them:

“For me, being a creature of habit, I would look for a device that would fit into my habits. And now you’re cutting down on the odds of not forgetting because you usually never forget your watch. It’s just part of your daily routine.” - Person with dementia #2

##### User friendly

To be user friendly, devices should have limited required steps and user manuals must be easily accessible and available. Many participants also noted that it is vital for locator devices to be as minimally complex as possible, so the person with dementia remembers how they work. Without such user-friendliness, users may forget how to use them.

“With my mom, I’ve given her a…cell phone…and she will forget the number, she’ll forget that she has a phone, or she’ll forget how to use it.” - Care Partner #2

##### Guidance and support

Care partners, persons with dementia and service providers indicated that for a device to be usable, it is necessary for users to receive instructions and troubleshooting information from the locator device companies. This guidance and support should be available, effective and responsive. If a user requires assistance, support should be available at all times to minimize ‘downtime’ and disruptions in its use. Participants also highlighted that locator device companies should take ownership of ensuring the device is effective. Without this, a person with dementia could falsely assume that the device is effective, and their safety is being managed when, in fact, they are at risk due to a device malfunction or service disruption. One participant with dementia explained that a company from whom she purchased a device and services went out of business and provided no indication of the discontinuation of services:

“Nobody has contacted me for a while about my device. I was trying to get hold of somebody, and nobody was answering, and I couldn’t get through to anybody… [I came to learn that] my device hasn’t been working for months and nobody told me …. I thought I was protected, people around me weren’t checking on me because they thought I was protected, and I was not.” - Person with dementia #3

#### Features

**Figure 3.**
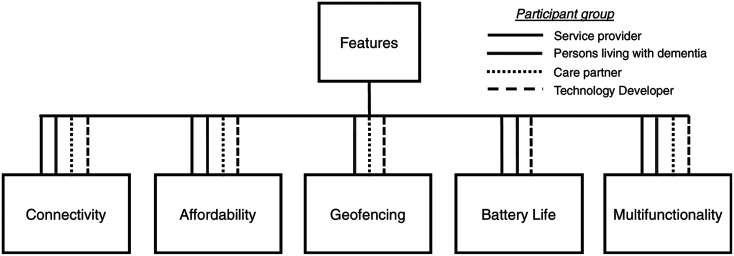
Factors that encompass the essential feature of locator devices for people with dementia, by participant group.

Six types of features were identified as factors influencing usability of locator devices in this category, namely: connectivity, affordability, geofencing, battery life and multifunctionality (Figure 3).

##### Connectivity

Connectivity is described as the strength of the connection that allows the transmission of the user’s location. Connectivity was identified as an important feature by all stakeholder groups. The precision of the location and coverage (i.e. access to cellular networks or WiFi) can significantly impact the time to find the missing person.

“We face challenges with the quality of data in downtown areas due to signal blockage where there are more high rises…this has led to issues among some of our clients when trying to get the exact location of a loved one”. – Service Provider #4

##### Affordability

Affordability refers to the costs associated with the use of a device. All groups identified cost as being a factor when choosing a device. Some devices include both an initial fee as well as monthly or annual service fees. Some care partners and persons with dementia have a fixed income which impacts how much they are able to spend on a locator device. Participants indicated that it is important to them that it is transparent how much a device will ultimately cost:

“The income is not as fluid as it was, and so you’re very fixed. So cost is a big deal.” - Care Partner #1

##### Geofencing

Developers, care partners and persons living with dementia indicated that geofencing is a beneficial feature of locator devices. Geofencing for the context of this study refers to a virtual perimeter for a real-world geographic area. A predefined boundary can be set where an alarm is generated when someone leaves this boundary. Participants noted that geofences should have an adjustable perimeter and may need to become smaller as the dementia progresses.

##### Battery Life

Service providers, developers and persons with dementia spoke of the importance of battery life when choosing a device. Not only does a device need to last long enough to ensure that the user can be located, but it must also last several days in case the user or care partner forgets to recharge it. To mitigate this challenge, the device should alert the person with dementia and care partner when the battery is low.

##### Multifunctionality

Multipurpose refers to locator devices having capabilities beyond locating missing persons with dementia. Participants with dementia indicated that multiple purposes for the locator device, such as being able to measure heart rate, blood pressure and activity level, is advantageous as it would streamline the number of devices that they and their care partners must contend with.

All stakeholder groups spoke about how the ability for the device to communicate within a circle of care chosen by the person wearing the device is a factor of its usability. If a device has two-way communication, the care partner and persons living with dementia can speak with each other through the device. This could help care partners to reassure the user when they are lost and provide instructions on next steps. Some locator devices also have a ‘help’ (or panic) button which alerts care partners or emergency responders when pressed. However, participants were not in agreement about the usefulness of the panic button; some care partners felt this feature was useless because the person with dementia would not remember that the button was available to them. Participants with dementia, on the other hand, viewed it as helpful way to communicate during emergencies:

“When you’re in the middle of the night or in the middle of somewhere you get panicked, and you can’t decide whom to call and what to do. So, if it’s just a button then you will know right away that you’re lost, and they [their care partner] will figure out where you are, and they can call you.” - Person with dementia #5

#### Physical properties

**Figure 4.**
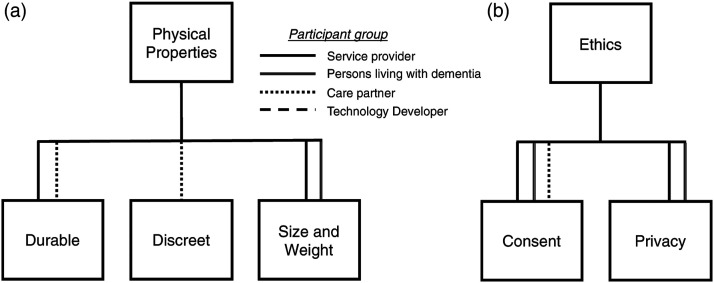
Factors that encompass: (a) physical properties, and (b) ethics on locator devices, for people with dementia, by participant group.

Physical properties of locating devices were identified as important factors by service providers, care partners and participants with dementia. This was described as how durable, discreet, heavy and large a device was (Figure 4(a)).

##### Durability.

Durability was described as how susceptible the device was to being damaged and needing repairs. Service providers and care partners highlighted the importance of the device to be robust, difficult to break when dropped, and waterproof. One of the care partners stated:

“If it’s on the wrist or even in a pendant, they can lean over the sink and get it wet. It has to be waterproof… And I noticed that they also get in the shower and don’t take them off.” - Care Partner #1

##### Discreet

All participants confirmed the importance of discreet device that is unnoticeable and does not draw attention to reduce stigma associated with such devices and with dementia. Some care partners indicated that not only would it be helpful for the device to be inconspicuous to the public but also to the person with dementia who may be hesitant to wear it.

“[My mom] doesn’t want to show people that she’s sick… it should be as hidden as possible… Many [people with dementia] are very stubborn. The less sometimes they know the better.” - Care Partner #2

##### Size and weight

Size and weight are both important attributes of devices as they determine how easy it would be for a person to wear the device all day. Service providers and participants with dementia both indicated it is important that the device is not too large or heavy as it may decrease the acceptance and usability.

“I had it [the GPS device] under my coat, it was big, and it was horrible that way. It was like wearing a big flashing sign.” – Person with dementia #5

##### Ethics

Ethics was highlighted as being a key factor by all stakeholder groups and consisted of consent and privacy (Figure 4(b)).

##### Consent

In this study, consent indicates that the person wearing the locator device is informed about the device and has agreed to wear it. While some care partners expressed that it was important for a person with dementia to consent to using the device, others believed that locator devices should be implemented regardless of their expressed wishes because the need to manage risks outweighed choices of a person with dementia. A care partner commented:

“[My husband] would have never given his consent even at the beginning – he liked the independence of making his own decisions and doing what he wanted. And I would have had to do it without his consent.” - Care Partner #1

##### Privacy

Privacy was considered as a key factor by all stakeholder groups, however, not all participants held similar views regarding its importance. Some service providers and participants with dementia noted that privacy was key. One participant with dementia stated that she did not want to share their information, and another stated that he would feel spied on:

“You’re going to get a lot of flak from people living with dementia because we are already feeling very threatened…and will feel like [we’re] being spied on” – Person with Dementia #2

Conversely, care partners and service providers indicated that privacy should not be an issue as the information would only be shared with individuals who have been chosen by the person using the device. Additionally, these participants indicated that they are ready to give up their privacy and ensure safety:

“But most of the time, the families, they’re willing to give up privacy if it means an increase in security for some.” – Service Provider #3

## Discussion

The purpose of this study was to identify the factors that have an influence on the acceptance and usability of locator devices among persons living with dementia, service providers, care partners and members of industry. To our knowledge, no standardized scale exists for assessing the acceptance and usability of locator devices for persons living with dementia and their care partners. Overall, criteria such as inclusivity, individualization, simplicity, features, aesthetic appeal and ethics were considered essential elements to the successful acceptance and usability of locator devices to mitigate the risks associated with individuals living with dementia at risk of getting lost and going missing.

Our recent literature review (Miguel Cruz et al., 2020) supports the development of a specific tool for assessing the acceptance and usability of locator devices for persons living with dementia and their care partners. This is the main motivation of conducting this study. For example, existing scales (e.g. SUS, ISONORM 9241/10, and Post-Study System Usability (PSSUQ) tested in persons living with dementia are not reliable and complex for users. The number of items is high (i.e. on average 15 items), with medium-to-high number of scale points (i.e. 5 points) and bidirectional response category labelling to rate the scale points (Miguel Cruz et al., 2020).

New theoretical models or approaches such as UTAUT (Venkatesh et al., 2003) UTAUT2 (Venkatesh et al., 2012), and the Non-adoption, Abandonment, Scale-up, Spread and Sustainability (NASSS) framework (Greenhalgh et al., 2017) incorporate a range of new dimensions to describe the acceptability and usability of technology. This is the case of subjective norm, hedonic motivation, habit, the value proposition, the social and contextual aspects of technology acceptance, technology adoption and usability. However, we believe other aspects of the understanding of the acceptance of technologies by older adults with dementia and their care partners are still missing. For example, important aspects such as personal autonomy and data privacy, the perception of feeling unsafe, quality of life, self-image, dignity and stigmatization, independence, activities and participation, occupation and security (safety) are equally important and have been absent in the usability and technology acceptance tools (Miguel Cruz et al., 2020).

In terms of inclusivity, there was expressed need from participants for including the voice of people with dementia in technology development and evaluation. Similar results were found in Neubauer, Lapierre, et al., 2018, where it was indicated among forum participants that people living with dementia are seldom included in the process of product design through to commercialization. Many individuals living with dementia want to be active agents in their own care, and problems, such as issues with adoption and usability, will arise when their voices are excluded (Deep, 2013; Alzheimer Society of Ontario, 2021). Desired features of locator devices, such as the interface design and type could be misunderstood or overlooked if the perspectives and preferences of persons with dementia are not sought. By involving people who have dementia and obtaining an understanding of their unique needs, satisfaction and subsequent adoption of such devices may increase (Lenker et al., 2013; Suijkerbuijk et al., 2019).

There were also concerns regarding the flexibility of the locator device so that it can be used despite the evolving needs associated with different stages of dementia. The lack of individual configurations of the device that can adapt to changing healthcare needs with advancing disease severity is one of the largest product characteristic limitations among locator devices (Freiesleben et al., 2021). While not yet included in existing locator devices, passive monitoring of an individual’s everyday activities, physiological status and emotional state via voice detection and sensors could also be used to predict functional changes which could be used to adjust the technology requirements and determine if additional services are required (Rogers & Mitzner, 2017).

The inherent need for individualization of locator devices due to the heterogeneity of persons living with dementia was also expressed by all participants in this study. Discrepancies between the needs of end-users and available products can discourage adoption Vermeer et al., (2019), which highlights the importance of not following a one-size-fits-all design approach (Freiesleben et al., 2021). Individualization among locator devices aligns with other existing usability scales, such as ISONORM 9241 (Prümper, 1997). Individualization to enhance the usability of locator devices can include three subcategories: (1) controllability (user is able to initiate and control the direction and pace of the interaction until the point at which the goal has been met), (2) conformity with user expectations (corresponds to the user characteristics, such as task knowledge, education and experience) and (3) suitability for individualization (interface software can be modified to suit the task needs, individual preferences and skills of the user) (Megges et al., 2017). Such characteristics, rather than external characteristics (i.e. technological experience or care partner burden), are significantly associated with usability ratings (Megges et al., 2017).

There was a consensus among study participants across all stakeholder groups that simplicity plays a significant role in the acceptance and usability of locator devices. Among simplicity includes the availability of guidance and support of the device. Customer service that includes product training, technical support (online or via. telephone), and product manuals are available offline is a central need of individuals living with dementia according to Meiland et al., (2014) and McCabe & Innes (2013). Devices that can seamlessly fit into the existing routine of people living with dementia and their care partners is also integral due to the already inherent risk of care partner burden that exists among this population (McHugh et al., 2012). Simplicity should be embedded in the design, and should include instruction and support for individuals living with dementia and their care partners (Dawe, 2006). This has the potential to improve the perception of surveillance technologies, such as locator devices, as being useful, which may lead to acceptance and use of a device (Juzwishin & Liu, 2015; Vermeer et al., 2019).

Regarding the features of locator devices, connectivity and multifunctionality, such as communication, were highlighted by all participants as being important. In terms of connectivity, due to the primary purpose of locator devices being to identify persons that are lost, precision of the coordinates of the person wearing the device is vital. This should take into consideration the coverage area found in rural and urban communities, in addition to the strength of the connection of the device. Products that do not provide reliable and accurate locations can lead to significant limitations in the time it takes to find the person wearing the device, which can subsequently lead to usage-related difficulties (Freiesleben et al., 2021). The integration of two-way communication between a user and care partners, and the availability of a panic button were also indicated as being valuable. Similar results have been highlighted by Liu et al. (2017) and Robinson et al. (2009). Such features can provide the care partner with a means of reaching out to a person with dementia to assist the user remotely in the event of a lost incident (Topfer, 2016).

Conflicting perspectives arose regarding the aesthetic appeal of locator devices, particularly related to device size. Participants with dementia and some care partners expressed that locator devices must be discrete. Freiesleben et al. (2021) also found that ‘less is more’ when it comes to the size of the device. Stigma remains common among persons who live with dementia due to the internalization of negative views of dementia (Alzheimer Society of Ontario, 2021). Locator devices that publicly indicate that the person lives with an impairment, such as large pendants around a individual’s neck (Megges et al., 2018), can have a significant influence on the user’s desire to wear the device when they are in their community (Neubauer & Liu, 2021).

At the same time, such discreet devices should not be used in a way that the person with dementia is unaware of the presence of the device. This is because hiding the device from the user does not uphold one’s right to autonomy and privacy (Wan et al., 2016). As explained by study participants, privacy is a significant concern among persons with dementia although their care partners may be less concerned about it. White and Montgomery (2014) also reported that their participants preferred smaller products to enable covert use of surveillance technologies with person who have dementia. The smaller the product, the better the compliance; in other words, persons living with dementia may be less aware that they are wearing a locator device when it is small and discreet (Niemeijer, 2015).

Yet, it is paramount to obtain informed consent from users who have dementia regardless of the stage of their dementia (Canadian Centre for Elder Law, 2019). The balance between devices that can support one’s autonomy but infringe on their personal privacy because the devices provide constant monitoring can result in ethical tensions. As noted by Yang and Kels (2016), these tensions may arise at the intersection of privacy, autonomy, dignity and consent. For example, a person’s need for autonomy and independent living and a care partner’s need to locate the person when lost might outweigh data security concerns (Niemeijer, 2015; Robinson et al., 2007). Ethical concerns go beyond autonomy-safety dichotomy (Yang & Kels, 2016). It also includes dignity and consent such as whether the device negates or worsens the stigma often associated with dementia; whether the care partner has formal legal designations to act as a surrogate decision maker for the person living with dementia; or whether the preferences and wishes of the device by the person living with dementia have been discussed. These tensions highlight the importance of confronting ethical, legal and policy considerations at the front end of product development and deployment to ensure new technologies, such as locator devices, are being used wisely and that their lifesaving potential is recognized (Yang & Kels, 2016).

Other ethical tensions elicited in this study was the false sense of security that locator device users can give end users. One person with dementia highlighted that using a locator device gave her a sense of security and confidence to continue to engage in ‘risky’ activities such as going for walks in the woods alone. Despite these feelings of confidence and security, she came to learn that the locator device company and service ceased to exist without notifying her. As a result, she spent months engaging in risky activities while using a device that was no longer functional. This false sense of security (Freiesleben et al., 2021), and the risks it presents was also described in other studies (Müller et al., 2017). Family care partners expressed concern about the dangers of susceptibility to errors, malfunctions, breakdowns and exposure to radiation, especially when using Global Positioning System (GPS)-based tracking devices with the dementia population (Müller et al., 2017). It is recommended that clear and transparent information about the potential risks of locator devices is shared with persons living with dementia and their care partners (Freiesleben et al., 2021). Individuals with dementia and care partners also benefit from education about the importance of having multiple strategies to reduce the risks associated with getting lost (Alzheimer Society of Ontario, 2021). Since each strategy will have some drawbacks and limitations, multiple strategies can compensate for one another. For example, wearing a MedicAlert® bracelet can supplement the use of a locator device.

Despite the increased need for proactive strategies, such as locating devices to keep persons living with dementia at risk of getting lost safe (Miguel Cruz et al., 2020), the usability and acceptance rates for these devices remain relatively low (Demers et al., 2016; Miguel Cruz et al., 2020; O’Sullivan et al., 2017). Exploring design opportunities and development methodologies for assistive technology can be a challenge, to mitigate this, as seen through factors identified in this study, an effective strategy could be to incorporate end-user and relevant stakeholder engagement throughout the cycle, promoting the outcome of creative solutions (Azad-Khaneghah et al., 2020; Meiland et al., 2017; Holthe et al., 2018; Lopes et al., 2016).

### Strengths and limitations of the study

The strengths of this study include its qualitative approach to allow for an in-depth exploration of a multifaced and complex topic (Morgan & Krueger, 1998; Stewart et al., 2007), as well as the inclusion of a range of stakeholders that are involved in the use of locator devices (i.e. service providers, persons living with dementia, care partners, technology developers). Another strength of this study was the flexibility used to engage care partners as research participants. Care partner burden was expressed among all participants which was exacerbated by the COVID-19 pandemic and public health restrictions that reduced the supports available to this group (Canevelli et al., 2020). We convened two small (i.e. 2–3 participants) focus groups to make the best use of their valuable time and provide flexibility in scheduling to ensure that their voices were heard.

The authors acknowledge two limitations of this study. First, only English-speaking participants who had no hearing, visual or severe cognitive impairments participated in this study. As a result, the findings reflect the experiences of only those persons who participated. Second, while locator devices are traditionally hardware, and more are becoming available in a combination of hardware and software such as smart phone mobile applications (e.g. Life360; https://www.life360.com/intl/). Although some participants referred to mobile application-based locator devices, it was not the focus of this study. As a result, not all of the findings will apply to mobile application-based locator devices.

## Conclusion

Persons living with dementia who become lost is a growing public health concern. To minimize the risks associated with getting lost and promote the adoption of proactive strategies to manage the risk, we identified relevant factors that have an influence on the acceptance and usability of locator devices. Such criteria were inclusivity, individualization, simplicity, features, aesthetic appeal and ethics. This work highlights the complexity and importance of including multiple perspectives of the usability of locator devices and the balance that needs to be achieved between a user’s autonomy, independence and safety. Future directions are to development of an acceptance and usability scale for locator devices, validate it with a wider user base, and disseminate the scale to community organizations and service providers.

## References

[bibr1-14713012211065381] Alzheimer Society of Ontario (2021). Finding your Way®. http://findingyourwayontario.ca.

[bibr2-14713012211065381] Azad-Khaneghah P. Neubauer N. Cruz A. M. Liu L. (2020). Mobile health app usability and quality rating scales: A systematic review. 16(7), 712–721. 10.1080/17483107.2019.1701103. 31910687

[bibr3-14713012211065381] Brittain K. Degnen C. Gibson G. Dickinson C. Robinson L. (2017). When walking becomes wandering: Representing the fear of the fourth age. Sociology of Health and Illness, 39(2), 270–284. 10.1111/1467-9566.12505. 28177148

[bibr4-14713012211065381] Canadian Centre for Elder Law (2019). Conversations about care: The law and practice of health care consent for people living with dementia in British Colombia. https://www.bcli.org/wordpress/wp-content/uploads/2019/02/HCC_report-Final_web_Mar-29-2019.pdf.

[bibr5-14713012211065381] Canevelli M. Valletta M. Toccaceli Blasi M. Remoli G. Sarti G. Nuti F. Sciancalepore F. Ruberti E. Cesari M. Bruno G. (2020). Facing dementia during the COVID-19 outbreak. Journal of the American Geriatrics Society, 68(8), 1673–1676. 10.1111/jgs.16644. 32516441 PMC7300919

[bibr6-14713012211065381] Cooper J. Burrow S. Pusey H. (2019). What are the perceptions of people living with dementia, family carers, professionals and other potential stakeholders to the use of global positioning systems to promote safer outdoor walking?: a qualitative literature review. Disability and Rehabilitation: Assistive Technology, 16(6), 614–623. 10.1080/17483107.2019.1686074. 31711328

[bibr7-14713012211065381] Dawe M. (2006). Desperately seeking simplicity: how young adults with cognitive disabilities and their families adopt assistive technologies. Proceedings of the SIGCHI Conference on Human Factors in Computing Systems - CHI ’06, New York, NY, 22–26 April, 2006. 1143–1152.

[bibr8-14713012211065381] Deep (2013). Collecting the views of people with dementia. www.dementiavoices.org.uk.

[bibr9-14713012211065381] Demers L Mortenson W. B. Fuhrer M. J. Jutai J. W. Plante M. Mah J. Deruyter F. (2016). Effect of a tailored assistive technology intervention on older adults and their family caregiver: A pragmatic study protocol. BMC Geriatrics, 16(1), 103–111. 10.1186/s12877-016-0269-3. 27177609 PMC4866430

[bibr10-14713012211065381] Eichler T. Hoffmann W. Hertel J. Richter S. Wucherer D. Michalowsky B. Dreier A. Thyrian J. R. (2016). Living alone with dementia: prevalence, correlates and the utilization of health and nursing care services. Journal of Alzheimer’s Disease, 52(2), 619–629. 10.3233/JAD-151058. PMC4927920 27031480

[bibr11-14713012211065381] Freiesleben S. D. Megges H. Herrmann C. Wessel L. Peters O. (2021). Overcoming barriers to the adoption of locating technologies in dementia care: A multi-stakeholder focus group study. Research Square, 21(1), 378. 10.21203/rs.3.rs-283981/v1. PMC8218472 34154542

[bibr12-14713012211065381] Furumiya J Hashimoto Y (2014). A descriptive study of elderly patients with dementia who died wandering outdoors in Kochi prefecture, Japan. American Journal of Alzheimer's Disease and Other Dementias, 30(3), 307–312. 10.1177/1533317514545826. PMC10852914 25115170

[bibr13-14713012211065381] Greenhalgh T. Wherton J. Papoutsi C Lynch J Hughes G A’Court C Hinder S Fahy N Procter R Shaw S (2017). Beyond adoption: A new framework for theorizing and evaluating nonadoption, abandonment, and challenges to the scale-up, spread, and sustainability of health and care technologies. Journal of Medical Internet Research, 19(11), e367. 10.2196/JMIR.8775. 29092808 PMC5688245

[bibr14-14713012211065381] Guba E. G. (1981). Criteria for assessing the trustworthiness of naturalistic inquiries. Educational Communication & Technology, 29(2), 75–91. 10.1007/BF02766777.

[bibr15-14713012211065381] Hadwen T Smallbon V Qing Zhang Q. D’Souza M (2017). Energy efficient LoRa GPS tracker for dementia patients. Proceedings of the Annual International Conference of the IEEE Engineering in Medicine and Biology Society, EMBS, Jeju Island, Korea, 11–15 July, 2017. 771–774. 10.1109/EMBC.2017.8036938. 29059986

[bibr16-14713012211065381] Hermans D. G. Hla H. U. McShane R. (2007). Non-pharmacological interventions for wandering of people with dementia in the domestic setting. Cochrane Database of Systematic Reviews, 2007(1), CD005994. 10.1002/14651858.CD005994.pub2. PMC6669244 17253573

[bibr17-14713012211065381] Holthe T. Halvorsrud L. Karterud D. Hoel K.-A. Lund A. (2018). Usability and acceptability of technology for community-dwelling older adults with mild cognitive impairment and dementia: A systematic literature review. Clinical Interventions in Aging, 13, 863–886. 10.2147/CIA.S154717. 29765211 PMC5942395

[bibr18-14713012211065381] Holtz L. Byrdsong Q. (2020). Applying ethical principles to enrolling older adults with cognitive impairment in research. Research Methods Cases: Medicine and Health. 10.4135/9781529716023.

[bibr19-14713012211065381] Hsieh H. F. Shannon S. E. (2005). Three approaches to qualitative content analysis. Qualitative Health Research, 15(9), 1277–1288. 10.1177/1049732305276687. 16204405

[bibr20-14713012211065381] Jotterand F. Lenca M. Wangmo T. Elger B. (2019). Intelligent assistive technologies for dementia: Clinical, ethical, social and regulatory implications. Oxford University Press. https://books.google.ca/books?hl=en&lr=&id=-F-pDwAAQBAJ&oi=fnd&pg=PP1&dq=Jotterand,+F.,+Ienca,+M.,+Wangmo,+T.,+%26+Elger,+B.+(2019).+Intelligent+Assistive+Technologies+for+Dementia:+Clinical,+Ethical,+Social,+and+Regulatory+Implications.+Oxford+University.

[bibr21-14713012211065381] Juzwishin D. Liu L. (2015). Usability of locator technology among home care clients at risk for wandering evaluation report. Albert Health Services

[bibr22-14713012211065381] Kim H. Sefcik J. S. Bradway C. (2017). Characteristics of qualitative descriptive studies: A systematic review. Research in Nursing and Health, 40(1), 23–42. 10.1002/nur.21768. 27686751 PMC5225027

[bibr23-14713012211065381] Lenker J. A. Harris F. Taugher M. Smith R. O. (2013). Consumer perspectives on assistive technology outcomes. Disability and Rehabilitation: Assistive Technology, 8(5), 373–380. 10.3109/17483107.2012.749429. 23350880

[bibr24-14713012211065381] Lincoln Y. S. Guba E. G. Pilotta J. J. (1985). Naturalistic inquiry. International Journal of Intercultural Relations, 9(4), 438–439. 10.1016/0147-1767(85)90062-8.

[bibr25-14713012211065381] Liu L. Miguel Cruz A. Ruptash T. Barnard S. Juzwishin D. (2017). Acceptance of global positioning system (GPS) technology among dementia clients and family caregivers. Journal of Technology in Human Services, 35(2), 99–119. 10.1080/15228835.2016.1266724.

[bibr26-14713012211065381] Lopes P. Pino M. Carletti G. Hamidi S. Legué S. Kerhervé H. Benveniste S. Andéol G. Bonsom P. Reingewirtz S. Rigaud A.-S. (2016). Co-conception process of an innovative assistive device to track and find misplaced everyday objects for older adults with Cognitive Impairment: The TROUVE Project. IRBM, 37(2), 52–57. 10.1016/J.IRBM.2016.02.004.

[bibr27-14713012211065381] Mangini L. Wick J. Y. (2017). Wandering: Unearthing new tracking devices. Consultant Pharmacist, 32(6), 324–331. 10.4140/TCP.N.2017.324. 28595682

[bibr28-14713012211065381] McCabe L. Innes A. (2013). Supporting safe walking for people with dementia: User participation in the development of new technology. Gerontechnology, 12(1), 4–15. 10.4017/gt.2013.12.1.006.00.

[bibr29-14713012211065381] McHugh J. E. Wherton J. P. Prendergast D. K. Lawlor B. A. (2012). Identifying opportunities for supporting caregivers of persons with dementia through information and communication technology. Gerontechnology, 10(4), 220–230. 10.4017/gt.2012.10.4.003.00.

[bibr30-14713012211065381] Megges H. Freiesleben S. D. Jankowski N. Haas B. Peters O. (2017). Technology for home dementia care: A prototype locating system put to the test. Alzheimer’s and Dementia: Translational Research and Clinical Interventions, 3(3), 332–338. 10.1016/j.trci.2017.04.004. 29067340 PMC5651437

[bibr31-14713012211065381] Megges H. Freiesleben S. D. Rösch C. Knoll N. Wessel L. Peters O. (2018). User experience and clinical effectiveness with two wearable global positioning system devices in home dementia care. Alzheimer’s and Dementia: Translational Research and Clinical Interventions, 4(2), 636–644. 10.1016/j.trci.2018.10.002. 30519629 PMC6260223

[bibr32-14713012211065381] Meiland F. J. Hattink B. J. Overmars-Marx T. De Boer M. E. Jedlitschka A. Ebben P. W. Stalpers-Croeze I. I. Flick S. van der Leeuw J. Karkowski I. P. Dröes R. M. (2014). Participation of end users in the design of assistive technology for people with mild to severe cognitive problems; The European Rosetta project. International Psychogeriatrics, 26(5), 769–779. 10.1017/S1041610214000088. 24507571

[bibr33-14713012211065381] Meiland F. Innes A. Mountain G. Robinson L. van der Roest H. García-Casal J. A. Gove D. Thyrian J. R. Evans S. Dröes R. M. Kelly F. Kurz A. Casey D. Szcześniak D. Dening T. Craven M. P. Span M. Felzmann H. Tsolaki M. Franco-Martin M. (2017). Technologies to support community-dwelling persons with dementia: A position paper on issues regarding development, usability, effectiveness and cost-effectiveness, deployment, and ethics. JMIR Rehabilation Assistance Technology, 44(11), E1e6376. 10.2196/REHAB.6376. PMC5454557 28582262

[bibr34-14713012211065381] Miguel Cruz A. Daum C. Comeau A. Salamanca J. D. G. McLennan L. Neubauer N. Liu L. (2020). Acceptance, adoption, and usability of information and communication technologies for people living with dementia and their care partners: a systematic review. Disability and Rehabilitation: Assistive Technology, 1–15). 10.1080/17483107.2020.1864671. 33378627

[bibr35-14713012211065381] Morgan D. L. Krueger R. (1998). Analyzing and reporting focus group results. SAGE Publications. https://books.google.ca/books?hl=en&lr=&id=Cl0e4o1rvWQC&oi=fnd&pg=PR13&dq=Krueger+RA.+Analysis+%26+reporting+focus+group+results.+Thousand+Oaks,+CA:+Sage+Publications,+Inc.%3B+1998.&ots=ytezFcRyPN&sig=xgP5_kcptUniBqAN2bq-0jayYhg&redir_esc=y#v=onepage&q&f=.

[bibr36-14713012211065381] Morse J. M. Barrett M. Mayan M. Olson K. Spiers J. (2002). Verification strategies for establishing reliability and validity in qualitative research. International Journal of Qualitative Methods, 1(2), 13–22. 10.1177/160940690200100202.

[bibr37-14713012211065381] Moser S. J. (2019). Wandering in dementia and trust as an anticipatory action. Medical Anthropology: Cross Cultural Studies in Health and Illness, 38(1), 59–70. 10.1080/01459740.2018.1465421. 29757676

[bibr38-14713012211065381] Moyle W (2019). The promise of technology in the future of dementia care. Nature Reviews Neurology, 15(6), 353–359. 10.1038/s41582-019-0188-y. 31073242

[bibr39-14713012211065381] Müller I. Mertin M. Ariane R. M. A. (2017). Technology as an area of conflict between autonomy and safety acceptance and attitudes of family caregivers in regard to technical assistance to ensure safe areas of movement for people with dementia diseases. ICT4AWE 2017 - Proceedings of the 3rd International Conference on Information and Communication Technologies for Ageing Well and e-Health, Porto, Portugal, 28–29 April, 2017. 2, 127–134. 10.5220/0006283001270134.

[bibr40-14713012211065381] Neergaard M. A. Olesen F. Andersen R. S. Sondergaard J. (2009). Qualitative description-the poor cousin of health research? BMC Medical Research Methodology, 9(Issue 11–5), 52. BioMed Central 10.1186/1471-2288-9-52. 19607668 PMC2717117

[bibr41-14713012211065381] Neubauer N. Hillier L. M. Conway C. Beleno R. Liu L. (2018b). Reflections of the use of locating technologies with persons with dementia: proceedings of a key stakeholder forum Neurodegenerative Disease Management, 8(3), 195–205. 10.2217/nmt-2018-0002. 29943695

[bibr42-14713012211065381] Neubauer N. A. Lapierre N. Ríos-Rincón A. Miguel-Cruz A. Rousseau J. Liu L. (2018a). What do we know about technologies for dementia-related wandering? A scoping review. Canadian Journal of Occupational Therapy, 85(3), 196–208. 10.1177/0008417418777530. 29972049

[bibr43-14713012211065381] Neubauer N. A, Liu L. (2021). Influence of perspectives on user adoption of wander-management strategies. Dementia, 20(2), 734–758. 10.1177/1471301220911304. 32164446

[bibr44-14713012211065381] Niemeijer A. (2015). Exploring good care with surveillance technology in residential care for vulnerable people. Universitair Medische Centra.

[bibr45-14713012211065381] O’Sullivan J. Nordheim J. Jordan L. Hesse B. Möller S. Antons J. (2017). Information and communications technology in dementia care: Acceptance among professional caregivers. Innovation in Aging, 1(suppl_1), 165. 10.1093/geroni/igx004.644.

[bibr46-14713012211065381] Patton M. (2002). Qualitative research and evaluation methods (Book, 2002). Sage Publications. [WorldCat.org] https://www.worldcat.org/title/qualitative-research-and-evaluation-methods/oclc/47844738.

[bibr47-14713012211065381] Peek S. T. Luijkx K. G. Rijnaard M. D. Nieboer M. E. Van Der Voort C. S. Aarts S. Van Hoof J. Vrijhoef H. J. Wouters E. J. (2016). Older adults’ reasons for using technology while aging in place. Gerontology, 62(2), 226–237. 10.1159/000430949. 26044243

[bibr49-14713012211065381] Poland B. D. (1995). Transcription quality as an aspect of rigor in qualitative research. Qualitative Inquiry, 1(3), 290–310. 10.1177/107780049500100302.

[bibr50-14713012211065381] Powell R. A. Single H. M. (1996). Methodology matters--v. International Journal for Quality in Health Care, 8(8), 499–504. https://academic.oup.com/intqhc/article/8/5/499/1843013. 9117204 10.1093/intqhc/8.5.499

[bibr51-14713012211065381] Price J. D. Hermans D. Evans J. G. (2001). Subjective barriers to prevent wandering of cognitively impaired people. Cochrane Database of Systematic Reviews, 2010(4), CD001932. 10.1002/14651858.CD001932. PMC8406984 11034735

[bibr52-14713012211065381] Prümper J. (1997). Der Benutzungsfragebogen ISONORM 9241/10: Ergebnisse zur Reliabilität und Validität (pp. 253–262). Vieweg+Teubner Verlag. 10.1007/978-3-322-86782-7_21.

[bibr53-14713012211065381] Robinson L. Brittain K. Lindsay S. Jackson D. Olivier P. (2009). Keeping In touch everyday (KITE) project: developing assistive technologies with people with dementia and their carers to promote independence. International Psychogeriatric Association, 21(3), 494–502. 10.1017/S1041610209008448. 19193255

[bibr54-14713012211065381] Robinson L. Hutchings D. Corner L. Finch T. Hughes J. Brittain K. Bond J. (2007). Balancing rights and risks: Conflicting perspectives in the management of wandering in dementia. Health, Risk and Society, 9(4), 389–406. 10.1080/13698570701612774.

[bibr55-14713012211065381] Rogers W. A. Mitzner T. L. (2017). Envisioning the future for older adults: Autonomy, health, well-being, and social connectedness with technology support. Futures, 87, 133–139. 10.1016/j.futures.2016.07.002. 28458395 PMC5407378

[bibr56-14713012211065381] Rosenberg L. Kottorp A. Nygård L. (2012). Readiness for technology use with people with dementia: The perspectives of significant others. Journal of Applied Gerontology, 31(4), 510–530. 10.1177/0733464810396873.

[bibr57-14713012211065381] Rowe M. A. Bennett V. (2003). A look at deaths occurring in persons with dementia lost in the community. American Journal of Alzheimer’s Disease and Other Dementias, 18(6), 343–348. 10.1177/153331750301800612. PMC10833970 14682082

[bibr58-14713012211065381] Sandelowski M. (2000). Focus on research methods: Whatever happened to qualitative description? Research in Nursing and Health, 23(4), 334–340. 10.1002/1098-240x(200008)23:4<334::aid-nur9>3.0.co;2-g. 10940958

[bibr59-14713012211065381] Shalev Greene K. Clarke C. L. Pakes F. Holmes L. (2019). People with dementia who go missing: A qualitative study of family caregivers decision to report incidents to the police. Policing: A Journal of Policy and Practice, 13(2), 241–253. 10.1093/POLICE/PAZ007.

[bibr60-14713012211065381] Stewart D. Shamdasani P. Rook D. (2007). Group dynamics and focus group research. In Focus Groups (pp. 19–36). SAGE Publications, Ltd. 10.4135/9781412991841.

[bibr61-14713012211065381] Suijkerbuijk S. Nap H. H. Cornelisse L. Ijsselsteijn W. A. De Kort Y. A. W. Minkman M. M. N. Baglio F. (2019). Active involvement of people with Dementia: A systematic review of studies developing supportive technologies, Journal of Alzheimer’s Disease, 69(4), 1041–1065). 10.3233/JAD-190050. PMC6597993 31156158

[bibr62-14713012211065381] Tilly J. (2015). Responding to the wandering and exit-seeking behaviors of People with Dementia. Journal of the American Geriatrics Society, 473–481.

[bibr63-14713012211065381] Topfer L.-A. (2016). GPS locator devices for people with dementia. In CADTH issues in emerging health technologies. National Center for Biotechnology Information. http://www.ncbi.nlm.nih.gov/pubmed/27809428. 27809428

[bibr64-14713012211065381] Venkatesh V. Morris M. G. Davis G. B. Davis F. D. (2003). User acceptance of information technology: Toward a unified view. MIS Quarterly: Management Information Systems, 27(3), 425–478. 10.2307/30036540.

[bibr65-14713012211065381] Venkatesh V. Thong J. Y. L. Xu X. (2012). Consumer acceptance and use of information technology: Extending the unified theory of acceptance and use of technology. MIS Quarterly: Management Information Systems, 36(1), 157–178. 10.2307/41410412.

[bibr66-14713012211065381] Vermeer Y. Higgs P. Charlesworth G. (2019 Jan–Dec). What do we require from surveillance technology? A review of the needs of people with dementia and informal caregivers. Journal of Rehabilitation and Assistive Technologies Engineering, 6, 2055668319869517. 10.1177/2055668319869517 31832230 PMC6891003

[bibr67-14713012211065381] Wan L. Müller C. Randall D. Wulf V. (2016). Design of a GPS monitoring system for dementia care and its challenges in academia-industry project. ACM Transactions on Computer-Human Interaction, 23(5), 1–36. 10.1145/2963095.

[bibr68-14713012211065381] Wherton J. Greenhalgh T. Procter R. Shaw S. Shaw J. (2019). Wandering as a sociomaterial practice: Extending the theorization of GPS tracking in cognitive impairment. Qualitative Health Research, 29(3), 328–344. 10.1177/1049732318798358. 30215572 PMC6380460

[bibr69-14713012211065381] White E. B. Montgomery P. (2014). Electronic tracking for people with dementia: An exploratory study of the ethical issues experienced by carers in making decisions about usage. Dementia, 13(2), 216–232. 10.1177/1471301212460445. 24599815

[bibr70-14713012211065381] World Health Organization (2020). Dementia. https://www.who.int/news-room/fact-sheets/detail/dementia.

[bibr71-14713012211065381] Yang Y. T. Kels C. G. (2016). Does the shoe fit? Ethical, legal, and policy considerations of global positioning system shoes for individuals with Alzheimer’s disease. Journal of the American Geriatrics Society, 64(8), 1708–1715. 10.1111/JGS.14265. 27394035

